# The effects of desynchronosis on the gut microbiota composition and physiological parameters of rats

**DOI:** 10.1186/s12866-019-1535-2

**Published:** 2019-07-12

**Authors:** Ksenia M. Klimina, Ekaterina G. Batotsyrenova, Roman A. Yunes, Elena H. Gilyaeva, Elena U. Poluektova, Taisia A. Kostrova, Anna V. Kudryavtseva, Maya V. Odorskaya, Vadim A. Kashuro, Artem S. Kasianov, Maksim B. Ivanov, Valery N. Danilenko

**Affiliations:** 1 Department of Genetics and Biotechnology, Vavilov Institute of General Genetics Russian Academy of Sciences, Moscow, 119991 Russia; 2 The Laboratory of Biochemical Toxicology and Pharmacology, Institute of Toxicology Federal Medical Biological Agency of Russia, Saint Petersburg, 192019 Russia; 3 Department of Molecular Biology and Genetics, Federal Research and Clinical Center of Physical-Chemical Medicine of Federal Medical Biological Agency, Moscow, 119435 Russia; 4 Laboratory of Post-Genomic Research, Engelhardt Institute of Molecular Biology, Russian Academy of Sciences, Moscow, 119991 Russia

**Keywords:** Biological rhythms, Desynchronosis, Circadian clock, Rats, Catecholamines, Protein expression, Gut microbiota, Antioxidant system

## Abstract

**Background:**

All living organisms experience physiological changes regulated by endogenous circadian rhythms. The main factor controlling the circadian clock is the duration of daylight. The aim of this research was to identify the impact of various lighting conditions on physiological parameters and gut microbiota composition in rats. 3 groups of outbred rats were subjected to normal light-dark cycles, darkness and constant lighting.

**Results:**

After 1 and 3 months we studied urinary catecholamine levels in rats; indicators of lipid peroxidation and antioxidant activity in the blood; protein levels of BMAL1, CLOCK and THRA in the hypothalamus; composition and functional activity of the gut microbiota. Subjecting the rats to conditions promoting desynchronosis for 3 months caused disruptions in homeostasis.

**Conclusions:**

Changing the lighting conditions led to changes in almost all the physiological parameters that we studied. Catecholamines can be regarded as a synchronization super system of split-level circadian oscillators. We established a correlation between hypothalamic levels of Bmal1 and urinary catecholamine concentrations. The magnitude of changes in the GM taxonomic composition was different for LL/LD and DD/LD but the direction of these changes was similar. As for the predicted functional properties of the GM which characterize its metabolic activity, they didn’t change as dramatically as the taxonomic composition. All differences may be viewed as a compensatory reaction to new environmental conditions and the organism has adapted to those conditions.

**Electronic supplementary material:**

The online version of this article (10.1186/s12866-019-1535-2) contains supplementary material, which is available to authorized users.

## Background

The adaptation of living organisms in their environments depends significantly on their ability to develop biological rhythms, which control the body functions starting from the cellular level up to behavior. According to the prevailing views of scientists today, the dark/light cycle plays a leading role in the organization of biosystems. The dark/light cycle is the main timekeeper regulating the development of endogenous circadian rhythms that influence almost all body functions. Daily fluctuations in biochemical processes affect every cell in the body. All metabolic reactions ranging from respiration, blood circulation, synthesis and secretion of hormones to the activity of the central nervous system have a rhythmic basis [1].

Eukaryotes possess innate periodic metabolic and behavioral programs. All these programs are based on self-sustaining biological oscillators with a period attached to the cycles of the environment they inhabit. The autonomy of the oscillator is supported by metabolic energy, therefore any changes in metabolism that lead to a change in rhythm at the cellular level, lead to a disruption in the oscillations and the system as a whole [2]. In order for the internal rhythms to stay in sync and not flow independently from each other, it is necessary to have a central “oscillator” that sets the pace for all the clocks in the body.

The main regulatory center of biorhythms – or the master clock – is located within the superchiasmatic nucleus (SCN) itself within the anterior hypothalamic region, and it is directly linked to the retina. It is the retina which allows the SCN to keep record of lighting parameters, such as the wavelength and the duration of exposure time. The SCN coordinates light-dark and sleep-wake cycles, as well as metabolic events that occur within the peripheral tissues [3]. The circadian oscillators are in sync not only with exogenous pacemakers, but also among themselves. Changes in hormonal concentrations in the plasma and the urine are a consequence of synchronized rhythmic oscillations in both the nervous and endocrine systems. The host circadian system controls a broad spectrum of physiological parameters [4].

It is known that one of the consequences of change in a cell’s functional state is the imbalance between the formation and neutralization of reactive oxygen species (ROS). A short-term increase in ROS could be a signal. The increase in ROS levels could alarm the cell to adapt to the changing environmental conditions. This short-term fluctuation of ROS levels is extinguished by the cell’s redox potential. However, when metabolic deviations occur, excessive production of ROS could lead to oxidative stress. Further development of oxidative stress expands the area of cellular damage reaching the cell’s membranes and membranes of organelles such as mitochondria, which leads to disruptions in a cell’s metabolism [5].

The stability of a biosystem and its ability to respond quickly to changes in the external environment are reflected in the concentration of hormones, in particular, catecholamines. It is established that feedback hormones are involved in the transcription of clock-proteins and their post-translational modification, which contributes to the formation and maintenance of rhythms [1]. The main proteins of the mammalian molecular clock can be divided into two groups: those expressed upon exposure to light (BMAL1 and CLOCK) and those expressed in the dark phase (PERs and CRYs). While the mRNA coding for BMAL, PER and CRY proteins experience oscillations in the SCN depending on the circadian phase, the concentration of CLOCK protein remains stable at all times. Other proteins regulating the circadian clock influence the expression of crucial genes, which are known as the clock-controlled genes [6].

The gut microbiota (GM) significantly influences the functioning of both the digestive and immune systems of the host [7, 8] and even affects the peripheral and central nervous systems via the gut-brain axis [9]. Previously the host molecular clock has been shown to influence the composition of the GM [10]. Studies on mice and humans have revealed that the composition and metabolic activity (energy intake, cell growth, DNA repair) of the microbiota (intestinal and oral) are also subject to daily changes [11, 12]. However, these changes are manifested only in certain taxonomic groups such as the family *Lachnospiraceae*, which has been identified as robustly rhythmic [13]. Oscillations in the microbiota are driven, in part, by timing of food intake, as well as by the transcriptional activity of the genes (Clock, Per1/2, Bmal1, Cry1/2). However, GM also exhibit their own endogenous circadian rhythms (CRs) [14, 15]. Disruption of the host molecular clock leads to changes in the GM composition, which in itself can become the cause of many diseases (oncologic, metabolic and neurodegenerative) [10, 14].

Desynchronosis is the disruption of biological rhythms caused by either endogenous or exogenous factors. It is a potential cause of many diseases such as cancer, infertility and psychiatric disorders. In mice, constant light causes abnormal circadian clock oscillations in the peripheral tissues, which are characterized by decreased amplitudes and a broad distribution of peak phases of the PER2 gene [6]. Experimental desynchronosis caused by constant exposure to light for 14 days activates systemic inflammation in rats and, in another study, was associated with a shift in enzyme oxidation-reduction in rat lymphocytes. Exposing rats to dim light during their dark phase leads to the disruption of circadian rhythms and metabolism. Housing rats in dim light conditions increases energy consumption up to 55% of total intake during the light phase compared with 36% in rats kept under standard light/dark phase conditions [16, 17].

In our experiment, we simulated desynchronosis in rats by subjecting them to abnormal dark/light cycles. The purpose of this paper was to study the effects of changes in the lighting regime on the rats' ability to adapt to the changing environmental conditions. Most of these parameters are indicators of homeostasis; therefore, we selected them to characterize the organism’s reactivity to different lighting conditions.

## Results

### Differential assay of urinary catecholamines

Catecholamines (CAs) are biogenic amines synthesized from the essential amino acid L-tyrosine. L-tyrosine uptake into the nerve terminal occurs via active transport; there it is converted through a series of reactions into L-DOPA, dopamine (DA), norepinephrine (NE) and epinephrine (E).

**Fig. 1:**
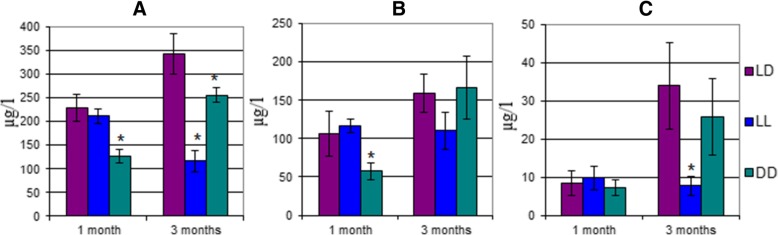
Catecholamine concentrations in rat urine after 1 and 3 months: **a**) Dopamine; **b**) Norepinephrine; **c**) Epinephrine. *– significant differences compared to LD1 (*p* < 0,05)

As compared to LD1, after 1 month of housing the rats under DD1, analysis of urinary CAs showed a significant decrease in DA (44,3%) and in NE (54%) but not in E (Fig. 1, Additional file 2: Table S1). None of the urinary CA levels were altered in LL1. As compared to LD3, after 3 months, the urinary levels dropped significantly in LL3: DA - 66%; E - 78%, but NE experienced no decrease. Urinary DA decreased significantly by 25% in DD3.

Housing the rats in constant darkness for 3 months (DD3) increased all urinary CAs (DA – 2-fold, NE – 3-fold, E – 3-fold) as compared to DD1. As for the group held in constant light, the levels of all CAs except DA did not change after 3 months as compared to LL1. It should be noted that after three months, the rats housed under normal light-dark cycles exhibited increases in their urinary CAs (DA – 50%; E – 300%).

### Indicators of lipid peroxidation and antioxidant activity in the blood

**Fig. 2:**
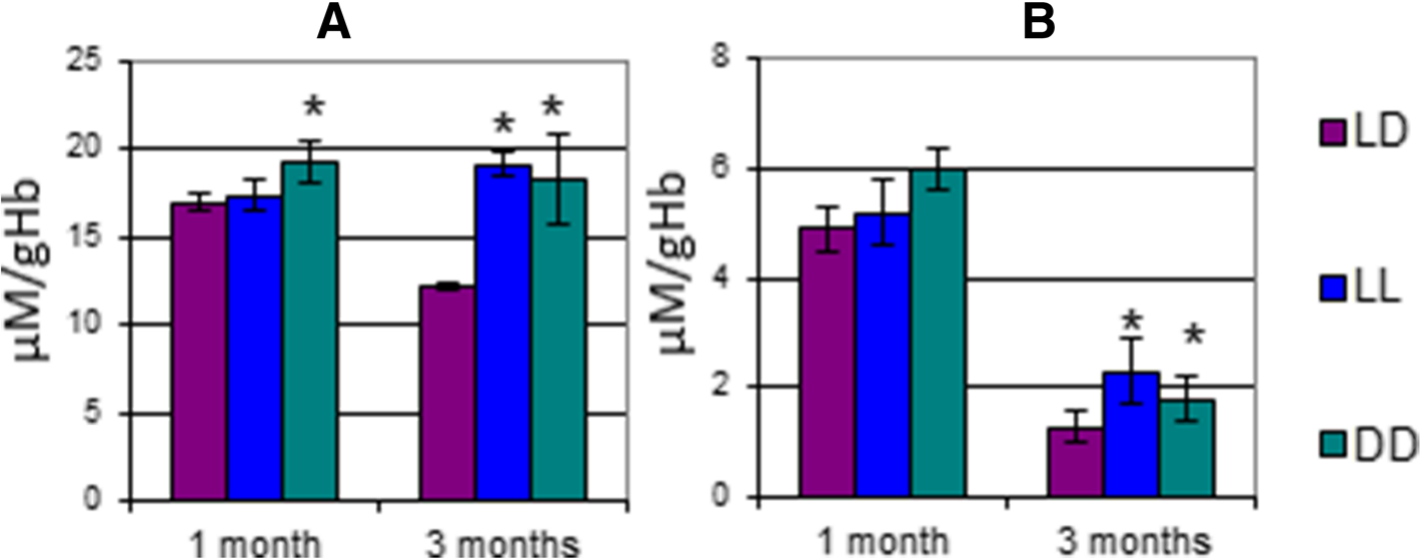
Antioxidant activity after 1 and 3 months: **a**) Reduced Glutathione; **b**) Glutathione S-transferase; **c**) Superoxide dismutase; **d**) Glutathione peroxidase; **e**) Glucose-6-phosphate dehydrogenase. *– significant differences compared to LD1 (*p* < 0,05)

After 1 month, the rats housed in constant light (LL1) as compared to the control group (LD1) showed: (i) a decrease of 25% in glutathione in the erythrocytes (Fig. 2, Additional file 2: Table S1); (ii) an increase of 57% in the enzymatic activity of glutathione S-transferase (GST); (iii) a decrease of 80% in the enzymatic activity of glucose-6-phosphate dehydrogenase (G6PD).

After 1 month, constant darkness (group DD1) as compared to the control group LD1 showed: (i) a small but statistically significant increase of 13.6% in malondialdehyde (MDA), a secondary product of lipid peroxidation; (ii) an increase of 87% in the enzymatic activity of GST and a decrease of 48% in the enzymatic activity of superoxide dismutase (SOD).

**Fig. 3:**
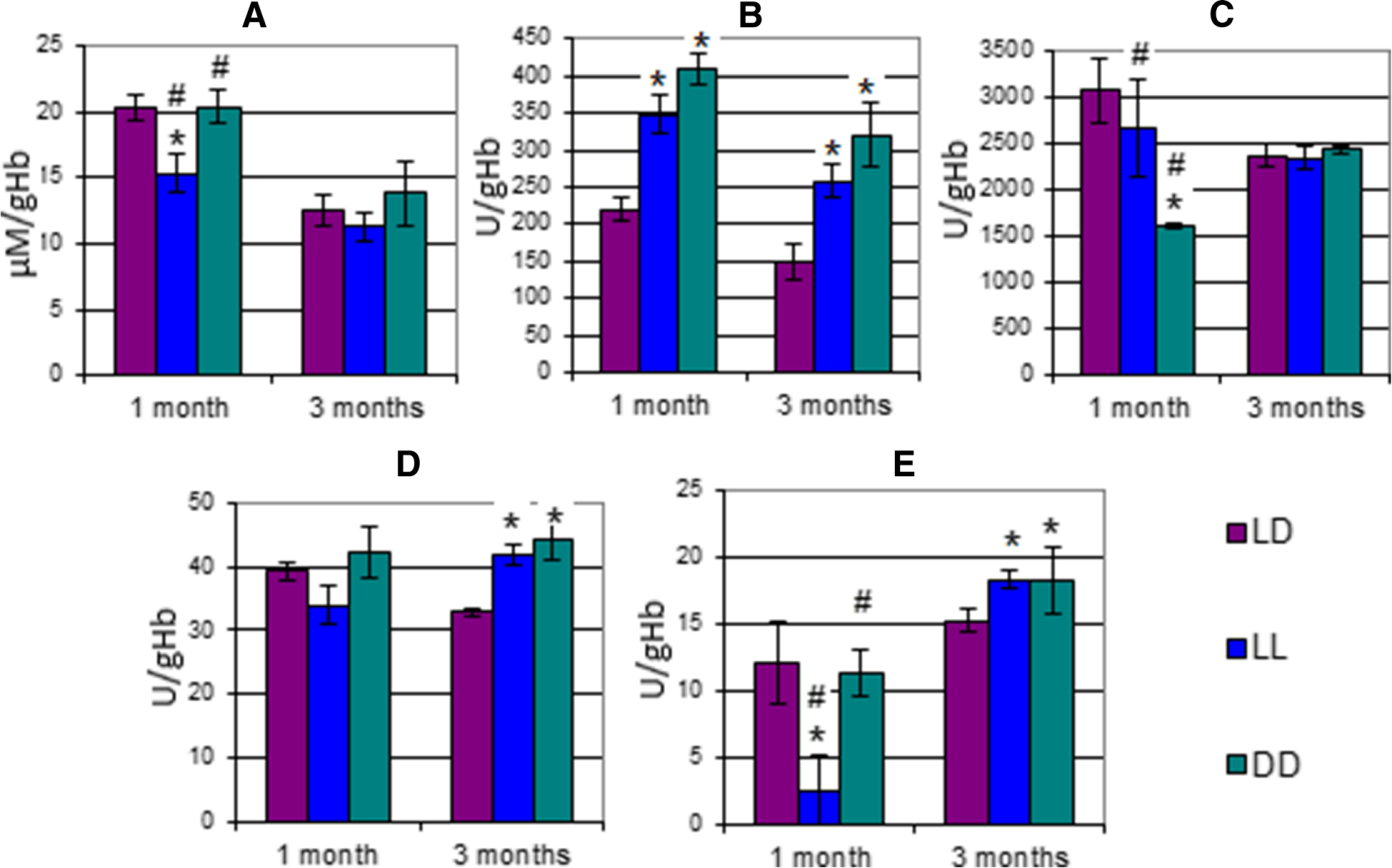
Lipid peroxidation products after 1 and 3 months: **a**) Malondialdehyde; **b**) Conjugated dienes. *– significant differences compared to LD1 (*p* < 0,05); # - significant differences between LL1 and DD1 (*p* < 0,05)

After 3 months: (i) MDA and conjugated dienes (CD) levels in LL3 increased by 58 and 77% respectively as compared to LD3 (Fig. 3, Additional file 2: Table S1). GST, glutathione peroxidase (GPx) and G6PD enzymatic activity in LL3 also increased by 71, 27 and 20% respectively; (ii) as for DD3, the rats showed a significant 51% increase in MDA levels and significant increases in the enzymatic activity of GST, GPx and G6PD, which reached 211, 33 and 20% respectively.

We also observed an overall decrease in lipid peroxidation and in the antioxidant defense systems data after 3 months as compared to 1 month in control groups.

### Disrupted biological rhythms impact on BMAL1, CLOCK and THRA concentrations

Western blot is one of the most efficient methods for performing a semi-quantitative analysis of differential protein expression. THRA is a nuclear orphan receptor in the SCN neurons. The CLOCK/BMAL heterodimer in light-deprived animals housed in darkness promoted the transcription of the *thra* gene, which confirms the presence of an intrinsic feedback loop independent of the light/dark cycle. The transcriptional repressor THRA inhibits the mRNA involved in regulating CRs in most rat tissues [6].

**Fig. 4:**
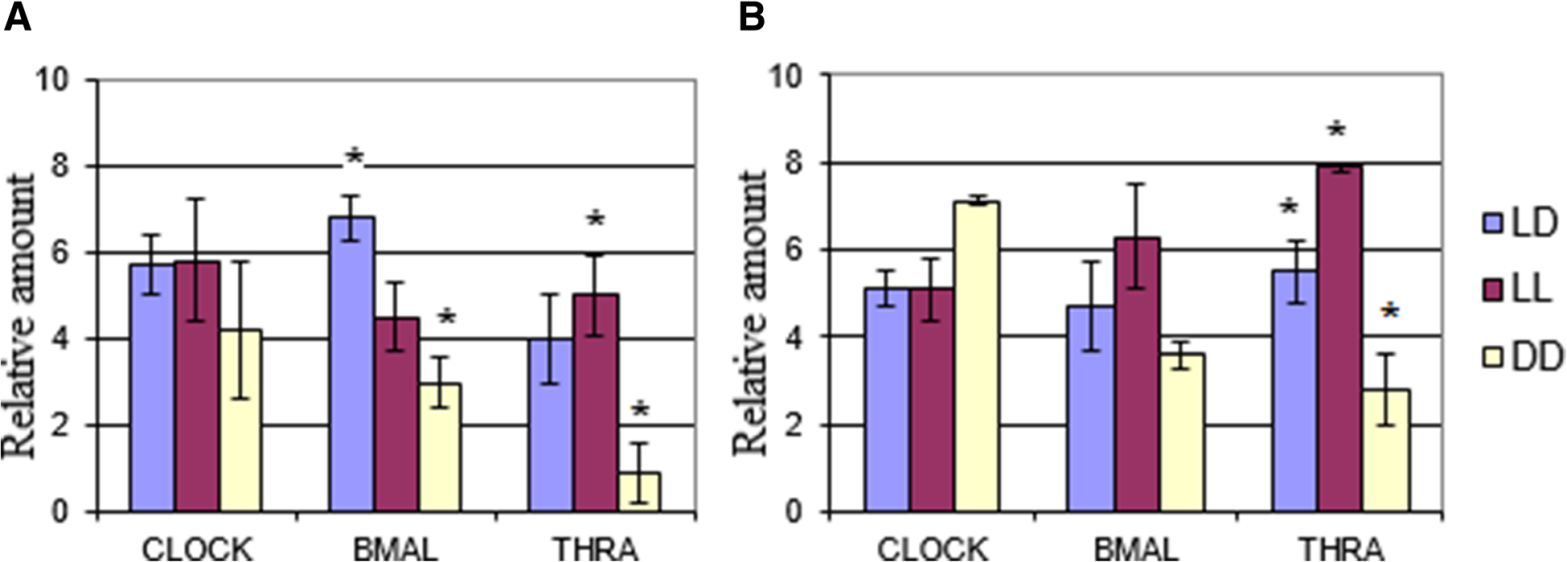
Relative amount of clock proteins in the hypothalamus: **a**) After 1 month; and **b**) After 3 months. *– significant differences compared to LD1 (*p* < 0,05)

After 1 month of housing rats in darkness, we found that the concentration of the protein BMAL in their hypothalamic structures dropped significantly (56%) as compared to the control group (Fig. 4). The rats housed in constant light also exhibited a significant decrease (34%) in Bmal protein concentrations as compared to the standard conditions. After 3 months, Bmal levels dropped by 23% in the constant darkness group while increasing by 34% in the constant light group as compared to rats housed in normal conditions.

After 1 month of housing rats in darkness, CLOCK protein levels dropped by 26% while remaining similar to those of the control group in the constant light group. We observed the same picture after 3 months, while CLOCK protein levels did not change in the constant light group, in the constant darkness group, CLOCK protein levels increased by 39% as compared to standard conditions.

In addition, after 1 month the concentration of the transcription factor THRA increased by 25% in LL1 and dropped by 77% in DD1. After 3 months, THRA levels increased by 50% in LL3 while decreasing by 45% in DD3 as compared to LD3.

### Different housing conditions and duration influence the composition of gut microbiota

**Fig. 5:**
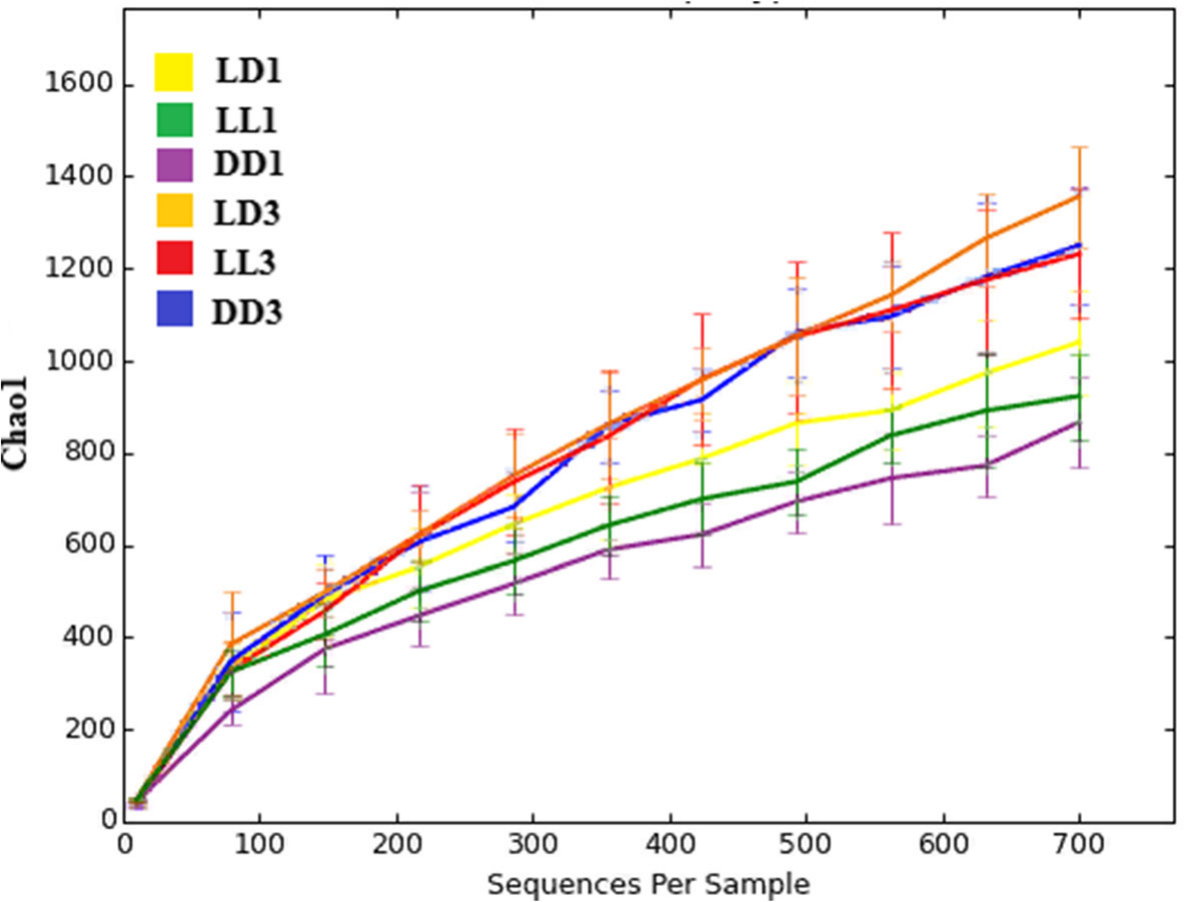
The impact of various housing conditions (constant light, constant darkness and normal conditions) on species diversity in rat GM

To determine the changes in GM composition, we performed a taxonomic analysis of the rat fecal microbiota. GM bacterial diversity was different between the groups of rats (Fig. 5). At point 1 month, the bacterial diversity in the experimental groups was lower than in the control group, with DD1 being the least divergent group. At point 3 months, the bacterial diversity increased significantly overall; for groups LL3 and DD3 it was similar, and yet, slightly lower than group LD3.

**Fig. 6:**
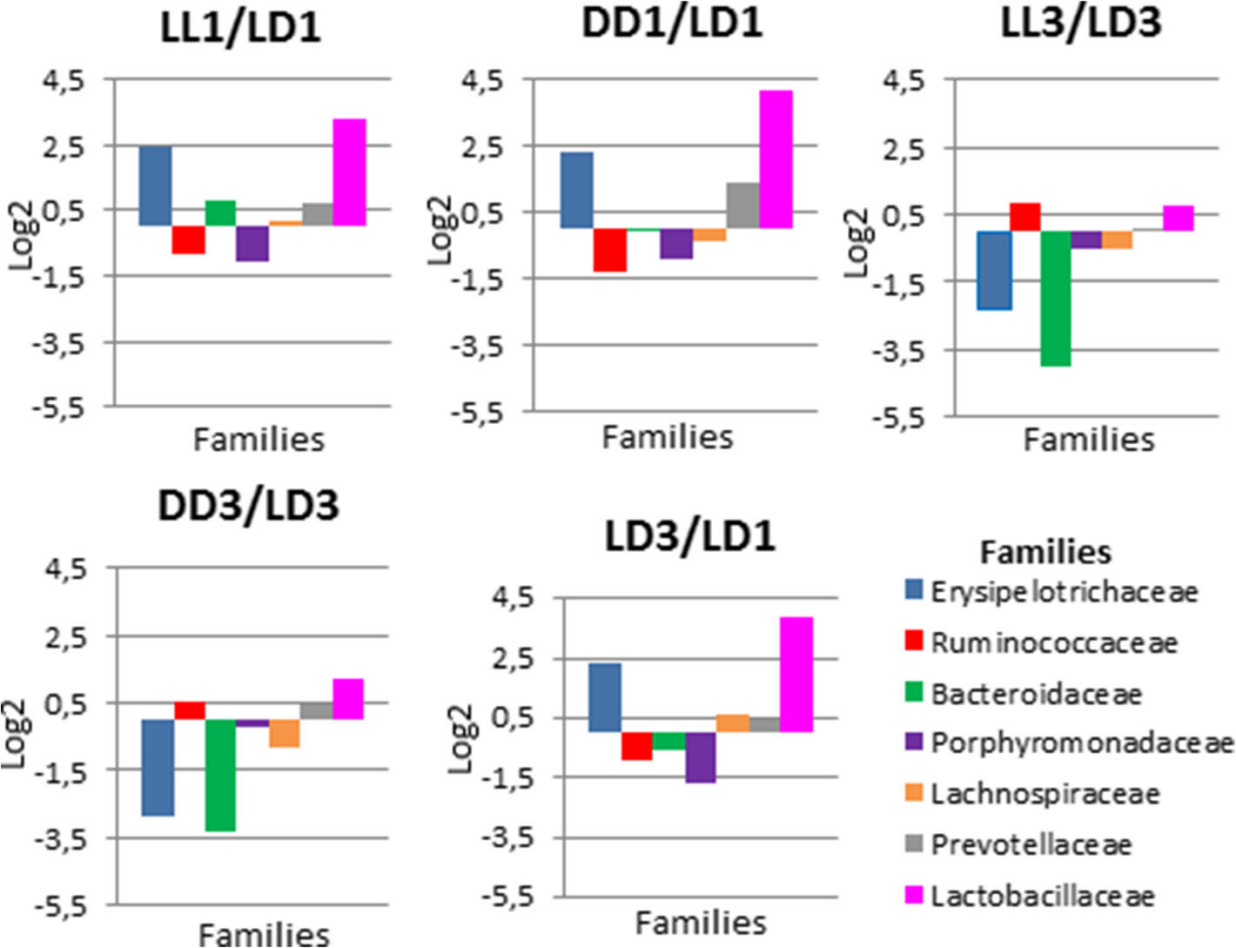
Ratios of the main bacterial families accounting for more than 1% of the metagenome in logarithmic scale

**Fig. 7:**
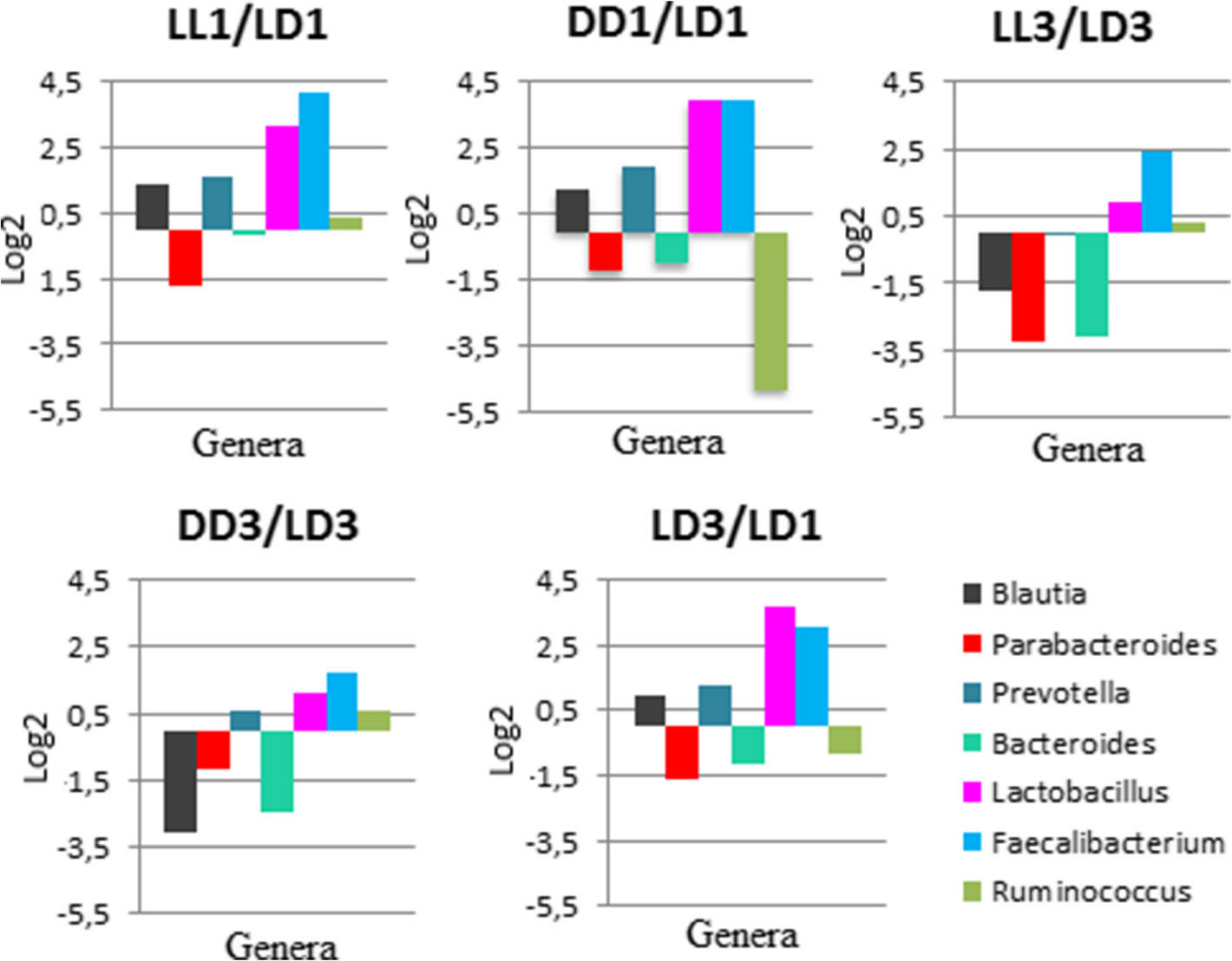
Ratios of the main bacterial genera accounting for more than 1% of the metagenome in logarithmic scale

The relative changes in the numbers of families and genera are represented in Figs. 6 and 7. In all cases, we observed a change in the relative number of families, including control groups when compared to each other (LD3/LD1). At point 1 month, the changes in groups LL1 and DD1 as compared to LD1 were very much similar, the most changes we observed in the families *Erysipelotrichaceae* and *Lactobacillaceae*; the increase in their ratio ranged between 4 and 5 and 8–16 times. These two families were also increased when LD3/LD1 were compared to each other. At point 3 months, the relative number of families was different; however, they were similar when the groups LL3/LD3 and DD3/LD3 were compared to each other. In these cases, we observed a decrease in the relative number of families *Erysipelotrichaceae* and *Bacteroidaceae* ranging between 4 and 5 and 7–9. Some families were not present in all groups: the family *Streptococcaceae* was present in LL1 and LL3 only in a small percentage (1.18 and 1.08% respectively) and was absent in the other groups. The family *Enterobacteriaceae* was present only initially in the control group LD1 (3.26%). The relative number of most of the genera also changed (Fig.7). The other genera that did not change between groups were: *Alistipes, Allobaculum, Alloprevotella, Butyrivibrio, Dorea, Helicobacter, Holdemanella, Intestinimonas, Oscillibacter, Phascolarctobacterium, Romboutsia, Saccharibacteria, Streptococcus*. After 1 month, the changes in the experimental groups LL1 and DD1 as compared to LD1 were similar; we observed an increase in the genera *Blautia*, *Prevotella*, *Lactobacillus* and *Bacteroides* and a decrease in the percentage of *Parabacteroides*; we also observed similar changes between the groups LD1 and LD3. However, there was a significant difference in the group DD1: the percentage of *Ruminococcus* as compared to LD1 decreased by 10 times.

At point 3 months, the ratio of the genera between the groups changed. The changes between the experimental groups LL3 and DD3 as compared to LD3 were also similar. We observed a slight increase in the ratio of *Lactobacillus* and *Bacteroides* and a decrease in the ratio of *Bacteroides* and *Parabacteroides*. Unlike the changes in point 1, we observed a decrease in the ratio of *Blautia*.

Although detected in the control group after 1 month, some genera disappeared completely from the experimental groups: *Alloprevotella*, *Helicobacter*, *Intestinimonas*, *Oscillibacter*, *Alistipes*. While the genus *Allobaculum* was present in all 3 groups after 1 month, we detected it only in the control group after 3 months. The genus *Rombustia* did not vary significantly among the groups and therefore can be considered as independent of lighting conditions.

### Desynchronosis lead to functional changes in the microbiota

**Fig. 8:**
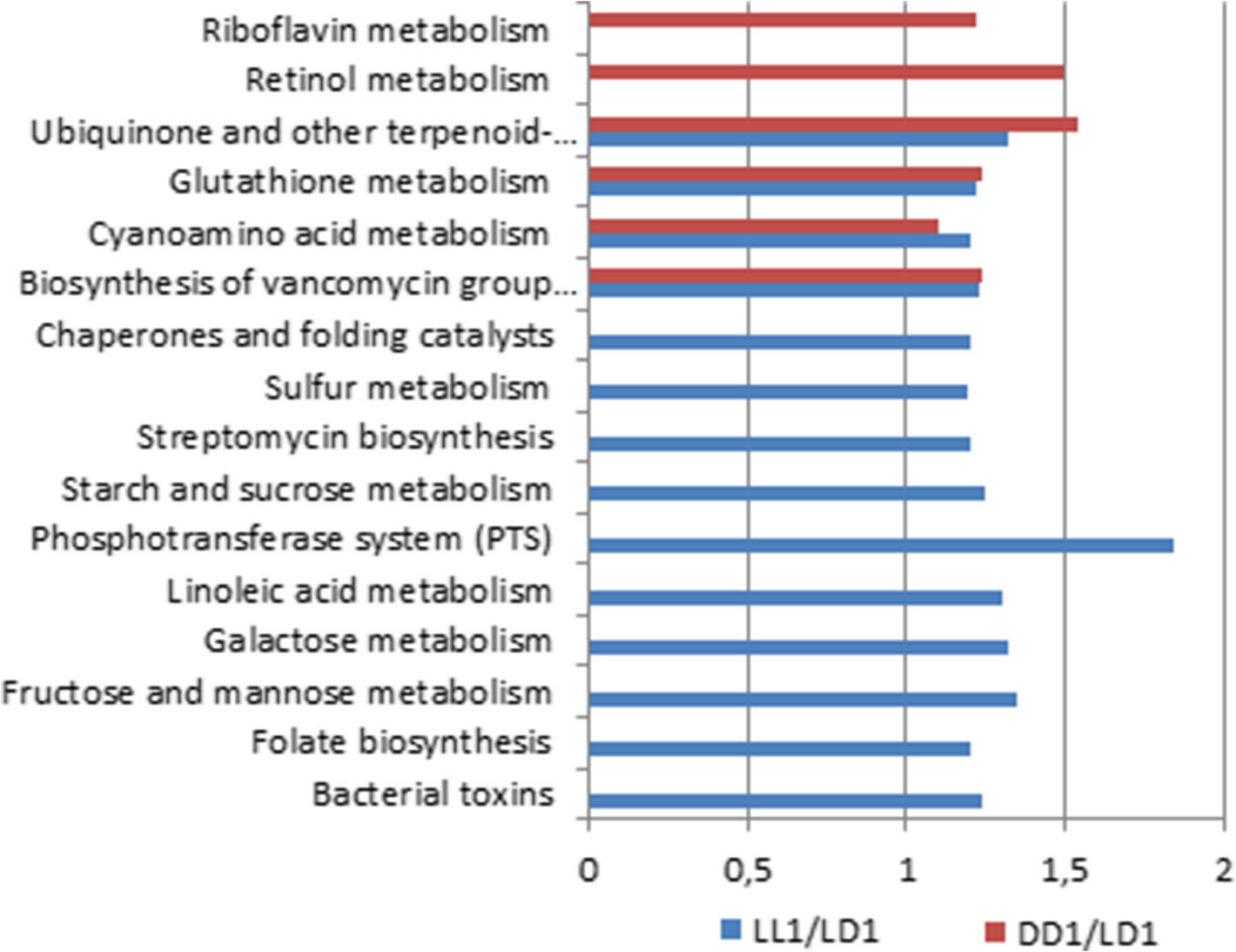
Functional properties of rat’s gut microbiota after 1 month of experimental conditions

We applied PICRUST, a method that infers the functional outputs based on 16S short reads to the obtained metagenomic data. Figure 8 represents those parameters showing statistically significant difference between the groups after one month. The GM functional capacity of rats in LL1 and DD1 altered both significantly and differentially as when compared to LD1. The experimental groups showed an increase in all functional potentials of rat GM. The LL1 group displayed the highest number of altered functions. The altered functional properties were involved in folate biosynthesis; sugar transport and metabolism; linoleic acid metabolism; streptomycin biosynthesis; sulfur metabolism; chaperones and folding catalysts; bacterial toxins synthesis. Retinol and riboflavin metabolism was altered only in DD1. Functional properties altered in both experimental groups were those involved in the biosynthesis of vancomycin group antibiotics; cyanoamino acid metabolism; glutathione metabolism; ubiquinone and other terpenoid-quinone biosynthesis. The increase in the functional properties listed above (LL1/LD1; DD1/LD1) was low (around 1.2-fold). The difference was much more tangible (1,35-1,84) for phosphotransferase system, fructose and mannose metabolism (LL1/ LD1), ubiquinone and other terpenoid-quinone biosynthesis, retinol metabolism (DD1/LD1).

## Discussion

The circadian clock is a regulator of many processes in the body including metabolism, immunity and behavior [18–23]. The GM contributes to many physiological processes and as recent data show, is no exception to circadian regulation [24–27].

In this study, we ran our experiments on rats. Due to their sociability, rats are frequently used as a standardized biological test system. They are nocturnal animals, and they adapt to unfavorable environmental conditions and colonize a wide variety of biotopes. Besides this, they remain active all year round and can also adjust their diurnal rhythmicity to the daily routine of the humans with whom they cohabit. According to Aschoff’s rule, diurnal animals kept in constant darkness stay awake for a much longer period of time than they sleep. Conversely, nocturnal animals exposed to constant light spend more time awake than asleep [28].

Rats exposed to constant light showed signs of anxiety and increased horizontal and vertical activities coupled with a strong interest in their environment. They also spent more time awake than asleep and gained approx. 20–30 g in weight per week. Rats housed in darkness were less active and sometimes aggressive. They spent more time asleep than awake and gained weight less efficiently, approx. 10–20 g per week (unpublished data).

The sympathoadrenal system plays a major role in the organism’s response to the changing conditions in the internal and external environments. The proximity of CAs to the sympathetic nervous system has functional significance for maintaining homeostasis in the body. CAs are formed in the brain and other nervous tissues where they act as neurotransmitters. Dopaminergic neurons are incapable of synthesizing NE due to their lack of dopamine-β-hydroxylase. The adrenal glands synthetize and store both E and NE in large quantities in secretory vesicles and are expelled out from the cell via exocytosis. NE is released from all postganglionic sympathetic nerve endings. This multistage process is regulated by the ability of the final products to inhibit the first enzyme involved in the initial stages of synthesis. Thus, DA, NE and E inhibit tyrosine hydroxylase. As these latter products accumulate in the neurons, a state of equilibrium is established when their synthesis and degradation rates become equal.

After 1 month of constant darkness, the amount of DA in the rats’ urine (DD1) decreased almost 2-fold. The level of NE decreased as shown in Fig. 1. As for E, its level in rat urine was no different from that of the control group (LD1). Changes in different light/dark cycles led to changes in the synthesis rate of E precursors and to the stockage of enough NE in vesicles to maintain homeostasis. This was due to night-time NE production, which activated epiphyseal adrenergic receptors, which in turn stimulated the secretion of melatonin accounting for the anti-stress effects. In this case E acts as a neuromodulator.

After 3 months of rats living in desynchronosis, DA, NE and E levels in the constant light group decreased as compared to the control group and the constant lighting group. The disturbance in melatonin synthesis (Constant light inhibits melatonin synthesis) and the development of hypoxia lead to a disruption in the cells energy balance which activated glycogenesis. Consequently, amino acids (Tryptophan, in particular, the precursor of serotonin and serotonin is the precursor of melatonin) are used to synthesize glucose. Since serotonin controls the activity of other neuromediators, alterations in brain serotonin levels causes an adrenal insufficiency. The depletion of adrenalin and its products leads to depression and a decrease in the organism’s adaptive reserves [29].

Constant darkness or constant light, as abnormal lighting conditions, both lead to hypoxia. As evidence for the rats developing hypoxia, we rely on the altered values of lipid peroxidation and on the altered activity of the antioxidant systems of erythrocytes. Lipid peroxidation is a process triggered by reactive oxygen species (ROS) which leads to the generation of lipid peroxides in the cells’ membranes; the lipid peroxidation-induced damage is normally repaired by specialized enzymes. However, the disruption of the established balance between pro- and antioxidants causes much more stable peroxidation products such as MDA to form. The basal MDA level in the body tissues is fairly low, thus any increase in its MDA levels is a potent indicator of lipid peroxidation and of a disbalance in the cells’ redox status. For the above reasons, we measured the MDA levels at 1 and 3 months. Another biomarker of intense lipid peroxidation is the level of CDs which had significantly increased in all the experimental groups by the end of the third month. These changes, which are triggered by hypoxia, are possibly related to changes in the fatty-acid composition of phospholipids of the cells’ membranes of erythrocytes.

Superoxide dismutase (SOD) is a potent enzymatic antioxidant that is considered to be the first line of defense against free radicals superoxide anion. SOD catalyzes the following reaction: **O**^**2−**^ **+ O**^**2−**^ **––––> H**_**2**_**O**_**2**_ **+ O**_**2**_. SOD is found in all oxygen-consuming cells. The rate of reaction catalyzed by SOD is extremely high and is limited only by the superoxide anion’s diffusion rate. SOD deactivates free radicals that may be generated either endogenously as a by-product of the electron transport chain in the mitochondria or exogenously as a result of an interaction with mixed-valent metals, ionized and UV radiation, ultrasound, etc. In our study, SOD activity dropped significantly after 1 month with a decrease of more than 50% in DD1.

The erythrocytes contain another important enzyme involved in antioxidation: selenium-containing GPx. This enzyme catalyzes the reduction of peroxides using a tripeptide called glutathione. G6PD is a key enzyme involved in the pentose phosphate pathway which plays a major role in maintaining the level of nicotinamide adenine dinucleotide phosphate (NADPH). NADPH supplies H^+^ to glutathione reductase which reduces glutathione disulfide (GSSG) to glutathione (GSH).

Based on the above, we can say that despite a disruption in the redox balance, the activity of the cells’ antioxidant systems does not indicate the presence of oxidative stress, a deteriorative process that causes irreversible destruction of macromolecules. In our case, the ROS serve as a trigger for regulating the bioenergetic processes. Previously, we demonstrated that a change in the dark/light cycle caused an energetic imbalance in the cellular metabolism as a consequence of hypoxia developing [29]. The ROS so generated can be used as activation energy and for modulating enzymatic activity. The ROS may also fulfill the role of an oscillator as a part of a pacemaker in the biochemical and physiological processes in body cells.

Little is known today about the role of hormones in the transcription and posttranslational modification of clock genes, but it has been suggested that clock proteins may be involved in the hormonal regulation of the cells’ metabolic reactions. Thus, the question of the hormones’ potential role in synchronizing circadian oscillators localized in different tissues of the body remains open for discussion. Unlike mammalian peripheral tissues and cultured fibroblasts which both harbor self-sustained molecular clocks, the master circadian clock governing behavioral rhythms is located in the hypothalamic SCN. In these cells, clock genes and their products form transcriptional/translational feedback loops where the proteins BMAL1 and CLOCK through E-box elements transactivate a series of genes, including *per* and *cry*. The translated PER and CRY proteins then suppress the function of the BMAL1–CLOCK complex [30, 31]. Posttranslational modifications such as phosphorylation regulate the activity of clock proteins (stability, localization and interaction) [32].

In our study, we established a correlation between Bmal1 concentrations in hypothalamic cells and CA concentrations in rat urine. After 1 month, DA and NE urinary concentrations dropped 2-fold in DD1 which correlates with a 2-fold decrease in Bmal1 concentrations as compared to LD1. After 3 months, we observed a significant decrease in all urinary CA levels in LL3. Bmal1 protein levels in the hypothalamus of the experimental groups were comparable to those in the control group. We suggest the hormonal system, in particular, CAs can be regarded as a synchronization super system of split-level circadian oscillators.

Proteins made from clock genes in most mammalian cells belong to a family of transcription factors containing a Per-Arnt-Sim (PAS) domain which is activated by light, oxygen, and other gases, as well as by steroid and peptide hormones (Additional file 1: Figure S1). Bmal1, the main molecular circadian oscillator, contains this light-sensitive domain. The PAS domain is also present in proteins (Cry, Per, Clock, Hif1α and Hif1β) and is involved in maintaining the cellular metabolic homeostasis. These facts allow us to hypothesize that such proteins participate in the transcription of hormone receptors.

While mRNA levels of BMAL, PER and CRY in the SCN are produced in a rhythmic fashion at specific times of the day, the expression of mRNAs coding for the CLOCK protein occurs constantly.

We also know that for the heterodimer CLOCK/BMAL1 to be functional (when Per and Cry are expressed) the acetylation of chromatin is necessary. Histone acetylation, deacetylation and methylation, which are rhythmically occurring processes, contribute significantly to the regulation of circadian rhythms. CLOCK protein, by participating in the acetylation of the dimer BMAL1, acts by itself as a histone-acetyltransferase.

It is possible that epigenetic changes in the cell’s genome drive the oscillation in BMAL1 and CLOCK proteins as the day is replaced by night.

Only few research papers studied the changes in composition and functional activity of the GM of animals housed in constant light or constant darkness. However, in those studies, the authors did not compare the differences between LL and DD groups and the duration of the experiments did not exceed 4 weeks [33, 34]. In our study, rats housed in both constant light and constant darkness exhibited significant changes in the GM composition. In the experimental groups LL and DD, we observed a decrease in alpha diversity of the GM as compared to the control group LD. There was also a significant change in the taxonomic composition of both LL and DD groups after 1 and 3 months of observation. The most dramatic changes were increases the genera *Lactobacillus* and *Faecalibacterium* (LL1/LD1, DD1/LD1, LD3/LD1) and decrease *Ruminococcus* (for DD1/LD1). After 1 and 3 months, the magnitude of changes in the GM taxonomic composition differed between the experimental groups (LL and DD) and the control group LD, but the direction of these changes was similar. Thus, the changes in the GM of rats, viewed as an adaptation process to the changing lighting conditions, were similarly oriented in both experimental groups. We note that the GM composition of the control group LD differed between the time points 1 and 3 months (LD1/LD3). This is possibly related to an ongoing process of adaptation of animals to the housing conditions.

As for the predicted functional properties of the GM which characterize its metabolic activity, they didn’t change as dramatically as the taxonomic composition. After 1 month, these changes were different between LL1/LD1 and DD1/LD1 groups. The number of significantly altered GM metabolic pathways between the rats housed in constant light (LL1/LD1) was much higher than that of rats housed in darkness (DD1/LD1). The amplitude of these changes however was small and did not exceed 1.2 in most cases. The most pronounced difference was in the phosphotransferase system between LL1/LD1 groups, which increased in LL1 by 1.8. The difference in fructose and mannose metabolism between LL1/LD1 groups was the second highest and it increased by 1.35. This increase in the phosphotransferase and fructose and mannose metabolism could be indicative of increase in overall metabolism of rats and their GM resulting from increased wakefulness. The increase in the metabolism of retinol in the GM of rats in DD1/LD1 is possibly due to a rise in the activity of the visual system of rats in the darkness.

After 3 months of the experiment, the differences in species diversity and functional capacity of the GM between the experimental and control groups were leveled out (Fig. 5). We believe that after 3 months the rats finally started adapting to their new environment.

## Conclusions

Changing the lighting conditions led to changes in almost all the physiological parameters that we studied. The obtained results showed the absence of oxidative stress after 1 and 3 months of experiment. Long-term exposure to altered external conditions, in our case lighting conditions, caused the oxidative system to perform a different function. The oxidative system in this case is responsible for mobilizing the organism’s defense systems and its preparation to endure other potential stressors. This phenomenon is known as «the adaptive oxidative stress response». Catecholamines can be regarded as a synchronization super system of split-level circadian oscillators. We established a correlation between hypothalamic levels of Bmal1 and urinary catecholamine concentrations. All differences may be viewed as a compensatory reaction to new environmental conditions and the organism has adapted to those conditions. Housing rats in both constant light and constant darkness influenced significantly the composition of the GM. There was a decrease in diversity and a significant change in the taxonomic composition of both LL and DD groups after 1 and 3 months of observation. The magnitude of changes in the GM taxonomic composition was different for LL/LD and DD/LD but the direction of these changes was similar. As for the predicted functional properties of the GM which characterize its metabolic activity, they didn’t change as dramatically as the taxonomic composition. The difference was much more tangible for phosphotransferase system, fructose and mannose metabolism, ubiquinone and other terpenoid-quinone biosynthesis, retinol metabolism. We believe that after 3 months the rats adapted to the new lighting conditions.

## Methods

All studies were conducted in accordance with the GSK Policy on the Care, Welfare and Treatment of Laboratory Animals and were reviewed and approved both by the *Institute of Toxicology Federal medical-biological Agency* of Russia (Bioethics Committee) and by the *Veterinary Department of St. Petersburg*.

Thirty-six male outbred rats from “Rappolovo” nursery (Saint Petersburg) aged 2 months were randomly divided into 3 groups and housed under 3 different light/dark cycles for 3 months: (i) LD - natural light condition - the control group (8–12 am, 1–8 pm – light; 8–12 pm; 1–8 am – darkness); (ii) LL – constant light (24-h light); (iii) DD - constant darkness (24-h darkness), (LD1, LL1, DD1 – after 1 month; LD3, LL3, DD3 – after 3 months).

The rats were placed in standard cages (*n* = 6) at 21-23 °C and were fed standard laboratory meals. Feces, blood and urine samples were collected after 1 month and 3 months. The study was carried out in a GLP-accredited laboratory.

### DNA extraction and 16S rRNA amplicon sequencing analysis

DNA was extracted from feces using MagNA Pure Compact Nucleic Acid Isolation Kit I (Roche, Germany) according to the manufacturer’s protocol for DNA_Bacteria. The lysis of bacterial cells was performed by MagNA Pure Bacteria Lysis Buffer following the recommended protocol for stool samples. The gDNA quantity was determined on the Qubit 2.0 Fluorometer (Invitrogen, USA) per manufacturer’s instructions.

Sequencing of the V3-V4 region of the 16S rRNA gene was performed following 16S Metagenomic Sequencing Library Preparation protocols. (https://support.illumina.com/downloads/16s_metagenomic_sequencing_library_preparation.html). Sequencing was performed using Illumina MiSeq Systems (Illumina, USA) with 2 × 250 bp paired-end runs following the manufacturer's instructions. We used the software trimmomatic v0.3 to trim the reads and FastQC v.0.10.128 to do some quality control. Raw sequence data were processed and analyzed using QIIME (Quantitative Insights Into Microbial Ecology, Version 1.9.1) software [35] and Ribosomal Database Project (RDP) http://rdp.cme.msu.edu/.

### Functional changes

We used PICRUST to predict the functional capacity of GM. PICRUST analyzes OTUs to predict the presence of biological pathways. The software is based on KEGG orthology groups (KOs) from the Kyoto Encyclopedia of Genes and Genomes (KEGG) databases [36].

Hierarchical clustering of samples was performed using R package Hclust (distance = euclidean, method = complete). Some samples were ruled out based on this clustering.

### Statistical analysis

Statistical analysis was performed using MS Excel. Independent t-test was applied for continuous variables. All significance tests were two-tailed; *p* < .05 deemed statistically significant.

### Catecholamines in urine

Urine was collected by placing the rats into individual metabolic cages. Urinary CAs were separated using a SHIMADZU liquid chromatograph on an Inertsil ODS-EP *column* (*Shimadzu*, Japan) with electrochemical detection for measuring concentration. Sample preparation was done using CAs in Urine kit (Chromsystems, Germany).

### Lipid peroxidation and antioxidant activity

We started by collecting whole blood in heparin-coated tubes (6 ml). In order to separate the plasma from the erythrocytes, we centrifuged blood at 3000 rpm and 4 °C for 10 min. Erythrocytes were washed 3 times with a cold physiological solution and recentrifuged. Hemolysis of erythrocytes was performed by adding a 5-mM Tris-HCl buffer, pH 7.6, to the cell suspension at a ratio of 1:9. Blood was then incubated at 4 °C for 30 min. The hemolysate was used in the subsequent assays in conjunction with reagent kits supplied by Randox (United Kingdom).

Concentrations of reduced glutathione in erythrocyte hemolysate were determined using 5,5′-di-thio-bis(− 2-nitrobenzoic) acid (DTNB) by the method of G. L. Ellman (1959) [37]. The activity of glutathione-S-transferase was determined by the method of W. H. Habig and W. B. Jacoby (1981) [38]. Concentrations of conjugated dienes in tissue homogenates and lysed erythrocytes were determined by the method of I. D. Steel (1977) [39]. The concentration of malondialdehyde was determined by the method of Uchiyama M. (1978) [40]. Hemoglobin in hemolysates of erythrocytes was determined using standard kits Ecolab (Russia).

### Western blot

We first isolated hypothalamic structures (MPO, ST-Ark, SHA) from rat brains and then topographically identified the relevant anatomical structures in the brain atlases [41]. We used the following buffer for sample lysis: 20 mM Tris (pH = 5.5), 150 mM sodium chloride, 1 mM EDTA, 1 mM EGTA, 1% Triton X-100 and with a protease inhibitor added just before homogenization. After homogenization, the samples were mixed with the following buffer: 2,42 g Tris hydrochloride, 6 g 6% SDS, 15 ml 15% Glycerin, 3 mg Bromphenolblue and 10 ml β-mercaptoethanol per 100 ml, pH = 6,7. Then the samples were boiled for 5 min at 95 °C. We separated equal amounts of protein using 10% sodium dodecyl sulfate-polyacrylamide gel electrophoresis (SDS-PAGE) and transferred them to nitrocellulose membrane (UltraCruz Nitrocellulose Pure transfer membrane sc-3718). The membranes were blocked for 1 h at room temperature using 3% nonfat milk and then incubated overnight at 4 °C with primary antibodies. Anti-rabbit IgG-peroxidase produced in goats (Sigma A0545-1 ml) and Anti-mouse IgG-peroxidase also produced in goats (Sigma A9044-1 ml) were used for identifying the BMAL and THRA proteins, respectively. The membranes were washed 3 times for 10 min each with Tris-buffered saline containing Tween (TBS-T) and then incubated with horseradish peroxidase-conjugated secondary antibodies for 1 h at room temperature. After further washing, the immunoreactive proteins on the membrane were visualized with enhanced chemiluminescence plus reagents (SuperSigna West Dura Extended Duration Substrate by Thermo Fisher Scientific). The intensities of the immunoreactive proteins were measured via computerized image analysis and normalized to β-actin.

## Additional files

Additional file 1: **Figure S1.** The impact of various housing conditions (constant light, constant darkness and normal conditions) on clock gene expression (CLOCK, BMAIL1, THRA). (TIF 466 kb)

Additional file 2: **Table S1.** Catecholamine concentrations, antioxidant activity and Lipid peroxidation products in rat. *– significant differences compared to LD1 (*p* < 0,05); # - significant differences between LL1 and DD1 (*p* < 0,05). (XLSX 11 kb)

## Data Availability

All data generated or analysed during this study are included in this published article and its supplementary information files. Sequence data (fastq files) (metagenomic sequencing data) were deposited in the NCBI’s SRA (Bioproject: PRJNA448813).

## Abbreviations

CAs: Catecholamines
CD: Conjugated dienes
CRs: Circadian rhythms
DA: Dopamine
DD: Constant darkness
E: Epinephrine
G6PD: Glucose-6-phosphate dehydrogenase
GM: Gut microbiota
GPx: Glutathione peroxidase
GSH: glutathione
GSSG: glutathione disulfide
GST: glutathione S-transferase
LD: natural light condition
LL: constant light
MDA: Malondialdehyde
NADPH: Nicotinamide adenine dinucleotide phosphate
NE: Norepinephrine
PAS: Per-Arnt-Sim
ROS: Reactive oxygen species
SCN: Superchiasmatic nucleus
SDS-PAGE: Sulfate-polyacrylamide gel electrophoresis
SOD: Superoxide dismutase
