# Supplementary figures and images for: The effects of desynchronosis on the gut microbiota composition and physiological parameters of rats
Source: BMC Microbiol. 2019 Jul 12;19:160. doi: 10.1186/s12866-019-1535-2 (PMC6626387; doi:10.1186/s12866-019-1535-2)

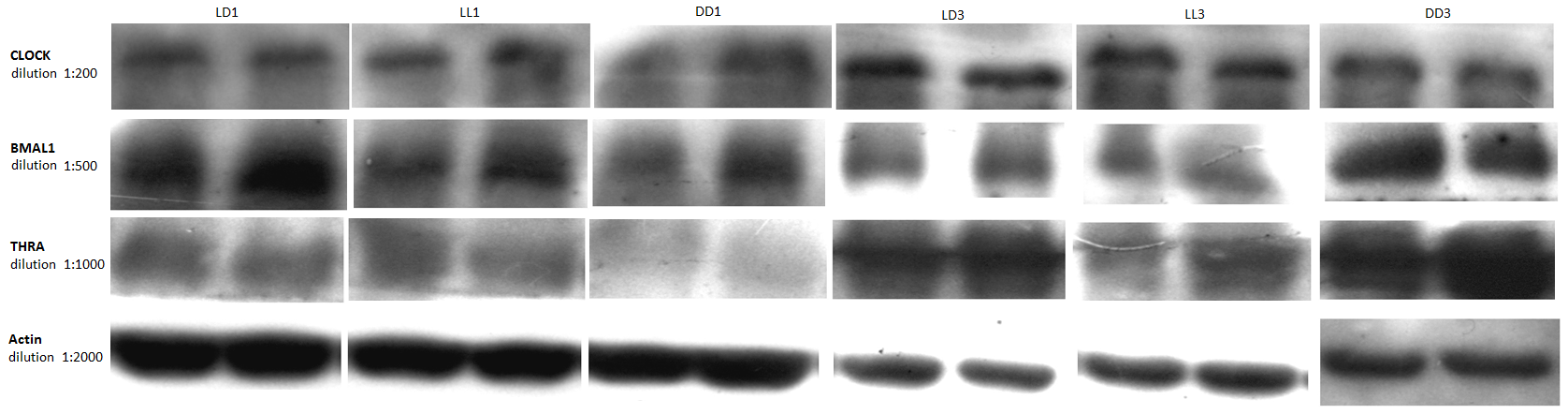

Supplement: Supplementary file 1 — Figure S1. The impact of various housing conditions (constant light, constant darkness and normal conditions) on clock gene expression (CLOCK, BMAIL1, THRA). (TIF 466 kb) [file 12866_2019_1535_MOESM1_ESM.tif]
